# BLAST screening of chlamydial genomes to identify signature proteins that are unique for the *Chlamydiales*, *Chlamydiaceae*, *Chlamydophila *and *Chlamydia *groups of species

**DOI:** 10.1186/1471-2164-7-14

**Published:** 2006-01-25

**Authors:** Emma Griffiths, Michael S Ventresca, Radhey S Gupta

**Affiliations:** 1 Department of Biochemistry and Biomedical Sciences, McMaster University, Hamilton, Ontario, L8N 3Z5, Canada

## Abstract

**Background:**

Chlamydiae species are of much importance from a clinical viewpoint. Their diversity both in terms of their numbers as well as clinical involvement are presently believed to be significantly underestimated. The obligate intracellular nature of chlamydiae has also limited their genetic and biochemical studies. Thus, it is of importance to develop additional means for their identification and characterization.

**Results:**

We have carried out analyses of available chlamydiae genomes to identify sets of unique proteins that are either specific for all *Chlamydiales *genomes, or different *Chlamydiaceae *family members, or members of the *Chlamydia *and *Chlamydophila *genera, or those unique to *Protochlamydia amoebophila*, but which are not found in any other bacteria. In total, 59 *Chlamydiales*-specific proteins, 79 *Chlamydiaceae*-specific proteins, 20 proteins each that are specific for both *Chlamydia *and *Chlamydophila *and 445 ORFs that are *Protochlamydia*-specific were identified. Additionally, 33 cases of possible gene loss or lateral gene transfer were also detected.

**Conclusion:**

The identified chlamydiae-lineage specific proteins, many of which are highly conserved, provide novel biomarkers that should prove of much value in the diagnosis of these bacteria and in exploration of their prevalence and diversity. These conserved protein sequences (CPSs) also provide novel therapeutic targets for drugs that are specific for these bacteria. Lastly, functional studies on these chlamydiae or chlamydiae subgroup-specific proteins should lead to important insights into lineage-specific adaptations with regards to development, infectivity and pathogenicity.

## Background

The *Chlamydiales *are clinically important intracellular parasites and endosymbionts of eukaryotic hosts, and cause a wide spectrum of diseases in humans and animals [1]. Recently, the taxonomic classification of this group was revised and currently contains at least 4 distinct families (viz.*Chlamydiaceae*, *Simkaniaceae*, *Parachlamydiaceae *and *Waddliaceae*) based on >90% 16S rRNA identity and a common developmental cycle [2,3]. While chlamydial infections are best known for the genitourinary, ocular and respiratory infections they cause in humans, the chlamydiae are also quite common in many wild and domestic animals with potential for severe zoonotic disease [4]. The newly described chlamydial species *Simkania *and *Parachlamydia *have also been found associated with human respiratory infections, while *Waddlia *has been implicated in abortion in bovines [5-7]. These revisions reflect important changes in perceptions regarding chlamydial diversity in line with recent discoveries of novel animal isolates (*Waddliae *and *Simkaniae*) and more strikingly of chlamydia-related endosymbionts or "environmental chlamydiae" (i.e. *Parachlamydiae*) infecting free-living amoebae [8-10]. Free-living amoebae, important components of soil and water ecosystems, are being increasingly recognized as vectors for various bacterial human pathogens [4,7,11,12].

Species are currently recognized as belonging to the *Chlamydiales *according to 16S and 23S rRNA similarities and pathogenic traits [2]. As these organisms are antigenically and genetically diverse, it is important to develop additional means to identify and distinguish them unambiguously from all other bacteria. The intracellular nature of chlamydiae species has been a hindrance in understanding their genetics, physiology and development. Hence, other means that can provide some insight in these regards are needed. In recent years, the genomes of several chlamydiae species have been sequenced including five *Chlamydiaceae *members (viz. *Chlamydia *(*Chl*.) *trachomatis, Chl. muridarum, Chlamydophila *(*Chlam*.) *pneumoniae, Chlam. caviae *and *Chlam. abortus *[13-16] and the environmental chlamydiae species *Protochlamydia amoebophila *(*Parachlamydiaceae*) [11]. Until recently when the *P. amoebophila *genome (formerly *Parachlamydia *UWE25;[17]) sequence was released, the information for chlamydiae-like organisms was practically non-existent, and it was virtually impossible to establish a core set of genes that are common to various *Chlamydiales *species. The *Protochlamydia *genome (2.41 Mb) was found to be about twice the size of various *Chlamydiaceae *species (1.04–1.23 Mb) and it showed a number of important differences including the presence of a complete TCA cycle and highly modified gene order [11]. The availability of these genomes has made it possible to carry out comparative studies. Horn et al. [11] reported 711 open reading frames (ORFs) or coding sequences that were shared among all chlamydiae genomes. However, many of these proteins have homologs in bacteria outside of the *Chlamydiales *and the proteins which were unique to only chlamydiae were not examined. Similarly, other studies which have determined proteins common to all *Chlamydiaceae *genomes also included peptides which were ubiquitous among bacteria [13-16]. While the work of Kalman et al. [18] revealed a number of potentially chlamydiae-specific genes, these studies were based on only 2 genomes, *Chlam. pneumoniae *and *Chl. trachomatis*, and the study was carried out when sequence information for other bacteria was limited.

Our recent work on comparative genomics is aimed at identifying novel and distinctive molecular characteristics of different groups of bacteria, including chlamydiae, which can be used for their identification, classification as well as genetic and biochemical studies [19-23] (see also [24]). We have recently described a number of molecular signatures consisting of conserved inserts and deletions (i.e. indels) in widely distributed proteins that are distinctive characteristics of all available chlamydiae species and are not found in any other bacteria [25]. In the present study, we describe a different kind of taxonomic marker, consisting of whole proteins that are specific for different groups of chlamydiae species, which provides additional powerful means for identifying these groups of species and for understanding their evolution as well as physiological characteristics. In this work, we have carried out systematic BLAST searches on all proteins or ORFs in the genomes of a number of chlamydiae species to compile profiles of proteins that are either uniquely shared by all *Chlamydiales*-species or particular subgroups (family or genera) within this phylum and can likely be used as distinct molecular markers for these groups. The study of these chlamydial group specific proteins should prove instrumental in the discovery of novel physiological characteristics that are uniquely shared by this important group of pathogens.

## Results

The present study was undertaken to identify unique proteins (or ORFs) which are found in the chlamydiae species at various taxonomic depths. Kalman *et al *[18] have previously examined the presence of chlamydiae-specific proteins in 2 sequenced genomes, *Chl. trachomatis *serovar D and *Chlam. pneumoniae*. Their study documented a number of proteins such as EUO, Gp6D, IncsB/C, LtuA/B, a number of Pmps and hypothetical proteins, which were specific for these species. However, because the number of species examined was so small, it was unclear how broadly these proteins were distributed among different chlamydiae species. At that time there was also no sequence information for the proteins from any chlamydiae-like organisms. Thus, based on this earlier study it was unclear whether the identified peptides were distinctive of different *Chlamydiales*, or only particular subsets of chlamydial species [18].

In order to identify chlamydiae-specific genes/proteins that are present at different phylogenetic depths, each ORF from the *Chl. trachomatis *and *Chlam. caviae *genomes were individually BLAST searched against all available databases. These species were chosen in order that sequences from both the *Chlamydia *and the *Chlamydophila *genera were represented in the searches. Results of the BLAST searches were inspected to identify proteins where either all of the observed hits were from other chlamydial species, or where all hits showing significant homology to the query protein (see Methods section) were members of the *Chlamydiales*. These studies have resulted in the identification of large number of signature proteins, which are uniquely found in chlamydiae species. Some of these proteins are present in all available chlamydial sequences indicating that they are likely specific for the entire *Chlamydiales *phylum. Other proteins were found to be specific for only the *Chlamydiaceae *family or the two genera, *Chlamydia *and the *Chlamydophila*, which comprise this family. Additionally, large numbers of proteins that are unique to *P. amoebophila *were also identified. Most of the identified chlamydiae-specific proteins or ORFs are annotated as hypothetical, hence their functions remain unknown. The genes for most of these proteins are scattered throughout the *Chl. trachomatis *and *Chlam. caviae *genomes, however a number of these hypothetical proteins were also found in clusters. Although, it is not clear whether these genes form operons, the presence of these genes in clusters suggest that they could be involved in related functions [26]. In the description of these proteins that follows, the "CT", "CC" and "PC" part of the descriptors refer to the source of the original query protein sequence from *Chl. trachomatis *(CT), *Chlam. caviae *(CCA) and *P. amoebophila *(PC) genomes, respectively.

### *Chlamydiales*-specific proteins

Proteins were considered *Chlamydiales*-specific if they were present in all sequenced *Chlamydia *and *Chlamydophila *genomes, as well as *P. amoebophila*, but were absent in all other bacteria. A total of 59 *Chlamydiales*-specific proteins were identified (Table 1). Of these, 49 peptides were annotated hypothetical indicating that these organisms have created new strategies allowing them to pursue a parasitic lifestyle with a minimum gene complement. Upon examination of the positions of various *Chlamydiales*-specific proteins within the *Chl. trachomatis *and *Chlam. caviae *genomes, some of them were found to be clustered on the chromosomes (those with adjacent CT numbers). A few of these gene clusters are discussed below.

Of the proteins annotated with a predicted function, only two represent outer membrane proteins (CT131 and CT546), which is surprising in light of large expansion of genes encoding chlamydial polymorphic membrane proteins [15,27]. One of these proteins CT546 is in a cluster with two other *Chlamydiales*-specific proteins (CT547 and CT548), whose functions are not known, but they could be involved in related functions [26]. Another important *Chlamydiales*-specific protein that is involved in the formation of their cell envelope is CT443 (OmcB). OmcB is not actually intercalated into a membrane. However, this protein is 4.4% cysteine, water-soluble and crosslinked to MOMP and another protein OmcA (CT444) to form the membrane complex [28,29]. OmcA and OmcB are two of the very few chlamydiae-specific proteins that have been functionally characterized [28,29]. The OmcA (CT444) protein is 15% cysteine and so has homology only to proteins with high cysteine content. It has a classic SPII signal sequence and is a proven lipoprotein [28,29]. OmcA and OmcB are transcribed together [28,29] and the proteins that are annotated as OmcA and OmcB are present in all genome sequenced chlamydiae [13-16], including *P. amoebophila *[11]. However, in BLASTp searches using either the *Chl. trachomatis *or *Chlam. caviae *OmcA (CCA00184) homologs as the query proteins (with or without the low complexity filter), an OmcA homolog was not detected in *P. amoebophila *although a protein PC0617 (accession number YP_007016) has been annotated as its OmcA homolog. Hence, by the criteria used in this study we regard CT444 as a *Chlamydiaceae*-specific and not a *Chlamydiales*-specific protein, and we have included it in Table 2. OmcA and OmcB annotation is presently based on the high cysteine content of the predicted protein products, as the cysteine residues are the basis for OmcA and OmcB function in Chlamydiaceae. Because OmcA and OmcB annotation has changed many times in the literature over the last 15 years [28,29], these proteins are best located by BLAST search. It should be mentioned that besides the *Chlamydiales *a homolog of CT443 with low E value (3e-25) is also found in *Rhodopirellula baltica *(Table 1). The *R. baltica *protein (accession number CAD72259) is larger in length (907aa) in comparison to the chlamydiae homolog and the sequence similarity between these two proteins is mainly seen in the C-terminal region. Importantly, the *R. baltica *homolog of OmcB contains no cysteine, hence the possible significance of the presence of this homologous protein in *R. baltica *(*Planctomycetes*) is presently unclear. A close relationship of chlamydiae to the *Planctomycetes*, which also lack peptidoglycan in their cell wall, has been noted in earlier studies [30,31]. Although such a relationship was not supported in a later study [32], in view of the presence of this commonly shared protein, it would be of interest to reexamine the relationship between these groups based on genomic sequences.

The finding of only a limited number of outer membrane proteins unique to various *Chlamydiales *suggests that the chlamydiae species most likely favour lineage-specific mechanisms or surface receptors for interacting with their different hosts rather than sharing a homologous system. Cell surface interactions are not limited to the outer membrane proteins. The LysM domain is a widespread protein module, which the available evidence suggests is a general peptidoglycan-binding module in cell surface proteins associated with cell wall degradation [33]. A LysM domain protein (CT474) was found to be common in all members of the *Chlamydiales *sequenced to date. The presence of this protein is intriguing as chlamydiae are not known to contain detectable levels of peptidoglycan (PG), despite housing genes for almost a complete PG biosynthetic pathway [34-36].

In addition to interacting with their hosts via cell surface molecules, the chlamydiae are also known to utilize a type III secretion system [37]. Genes encoding the basic type III secretory apparatus have been identified in all sequenced *Chlamydia, Chlamydophila *and *Protochlamydia *genomes [11,13-15]. These surface projections are used to inject chlamydiae-derived proteins into the host cytosol from within the inclusion body [38]. Orthologs or paralogs to effector proteins commonly found in other bacteria utilizing this apparatus have not been identified in chlamydial systems although they are likely to be critical for transducing the signals required to modulate host cell function. A type III secretion chaperone (CT274) was identified in all *Chlamydiales *members, which may facilitate a number of processes such as adhesion, cell signaling, transport and the perpetuation of infection. Another *Chlamydiales*-specific protein, CT273, was found in a tight cluster with CT274. The function of CT273 is not known, but it could also play a role in type III secretion [26]. Both of these genes lie in close proximity to CT271, another *Chlamydiales*-specific hypothetical protein which resides in the chromosome in the opposite orientation of the above mentioned ORFs.

Due to the reduction in metabolic capabilities of the chlamydiae, it is likely that many different permeases would be necessary for acquiring sufficient levels of different substrate molecules [18,39]. Members of the YjgP/YjqE family are predicted integral membrane proteins containing 6 transmembrane regions which are predicted to function as permeases [40]. Although YjgP/YjqE proteins are distributed throughout the major domains of life, a novel protein which is indicated to be related to this family, CT838, is uniquely found in various *Chlamydiales*. The iron-sulfur protein ferredoxin (Fd) is an electron acceptor which participates in the redox-based metabolisms in plastids, mitochondria and bacteria [41]. A novel predicted ferredoxin is uniquely present in various *Chlamydiales *species (CT312) suggesting that it serves a common evolutionary adapted function in these groups of species.

Another *Chlamydiales *specific protein, EUO (CT446), encoded by the *euo *gene (early upstream open reading frame) is found just upstream of the 2 genes encoding lysine-rich proteins Hc1 and Hc2 which are highly similar to the eukaryotic H1 histone [42,43]. One study has shown that Hc1 binds to DNA, inducing nucleoid compaction observed late in the chlamydial developmental cycle when vegetative reticulate bodies differentiate into the metablocially inert infectious particles known as elementary bodies [42]. The EUO protein, which is expressed early in the chlamydial cycle, has been shown to specifically cleave the C-terminal portion of Hc1, initiating dissociation of DNA-Hc1 complexes and DNA decondensation [42-44]. Little is known about the signals that trigger the different events throughout the chlamydial life cycle. However, the unique presence of the EUO protein (CT446) in various *Chlamydiales *species suggests a common mechanism for chromatin remodeling.

The *Chl. trachomatis *locus CT583 encodes Gp6D, a genomic paralog (31% similarity) of a plasmid-born virulence factor pGp6D [45]. Three of the four sequenced *Chlamydiaceae *species (except *Chlam. abortus*) [2] have been found to contain extrachromosomal DNA elements (cryptic plasmids) believed to encode genes which might play a role in pathogenicity and/or modulating virulence [46]. *P. amoebophila *does not contain an extrachromosomal plasmid. The chlamydial plasmids encode 8 ORFs of significance (>100 amino acids), and although 5 have been characterized, the function of pGp6D is unknown [45]. While the plasmid-borne pGp6D is only 102aa long, the genomic Gp6D homolog found in all *Chlamydiales *is more than twice that size (263aa). The high degree of similarity among plasmids from both *Chlamydia *and *Chlamydophila *species suggests that an ancestral plasmid was acquired by the chlamydial lineage, perhaps before divergence of the two *Chlamydiaceae *genera. That plasmid-less chlamydiae are rare suggests that plasmid loss is selected against – that the plasmids do have a function specific to this family, which is apparently absent in chlamydiae-like organisms. All of the proteins in this category, which are uniquely present in various *Chlamydiales*, have likely evolved in the last common ancestor of all chlamydiae and were subsequently passed on to various descendent species through vertical descent.

### *Chlamydiaceae*-specific proteins

The proteins in this grouping corresponded to those which were found strictly in species belonging to members of the *Chlamydia *and *Chlamydophila *genera, but whose homologs were not detected in *P. amoebophila*. Interestingly, among this category of proteins, 12 distinct gene clusters were located (described below). Of the 79 *Chlamydiaceae*-specific proteins identified in this work (Table 2), 60 were hypothetical. Those with predicted function are discussed below.

Chlamydiae possess an intracellular developmental cycle defined by the orderly interconversion of infectious, metabolically inactive elementary bodies (EBs) and noninfectious, dividing reticulate bodies (RBs) [1]. Only a few stage-specific genes are known, including the two late-stage genes encoding histone-like proteins. While chlamydial Hc1 (histone H1-like protein) is highly conserved (CT446), Hc2 (a second H1-like protein) is less conserved and variable in size (CT046) [42]. While both proteins are thought to participate in DNA compaction, Hc1 is found only in members of the *Chlamydiales *while Hc2 is found uniquely in the *Chlamydiaceae*, to the exclusion of *Protochlamydia*. Indeed, there are no other homologs of this protein outside of the *Chlamydiaceae *found in BLAST searches using *Chl. trachomatis *as the probe. LtuA and LtuB (CT377 and CT080 respectively) are two other proteins, which are uniquely shared by members of the *Chlamydiaceae *family. Both of these proteins are not expressed until the RBs begin to reorganize to EBs. These molecules do not bear strong homology to any other known protein and their functions are not known. The *ltuB *gene exhibits unusual stability properties in that the 5' end appears to break down, while the 3' end accumulates as a stable fragment of about 240 bases [47]. It has been hypothesized that the shorter *ltuB *RNA functions in someway in the late stage of the chlamydial developmental cycle [47]. These unique *Chlamydiaceae *specific proteins are likely playing distinctive stage-specific roles in the developmental cycles of the *Chlamydia *and *Chlamydophila *genera. The *ltuB *gene is found in close proximity to a cluster containing two other hypothetical *Chlamydiaceae*-specific proteins (CT082 and CT083).

A vast array of *Chlamydiaceae*-specific membrane proteins have been identified in our study, including Major outer membrane protein (MOMP or OmpA, CT681), polymorphic membrane protein PmpA (CT412) and PmpB (CT413) which lie in a cluster, the putative outer membrane proteins OmpD (CT812), OmpE, OmpF, OmpG, OmpH (CT869-CT872, which are clustered together on the *C. trachomatis *chromosome) and OmpI (CT874). Several of these predicted outer membrane proteins have recently been shown to be translated and localized to the surface of the chlamydial outer membrane [27,48]. Outer membrane proteins of microbial pathogens serve essential roles in engaging the host environment and can be important immunotherapeutic targets [49]. Besides the above outer membrane proteins, several other members of the outer membrane complex were uniquely present in all *Chlamydiaceae *species. Some of these e.g., porin-b(CT713) or SRP (previously annotated as CrpA) (CT442) [50], have been described as being immunogenic. PorB (CT713) is the target of neutralizing antibody responses *in vitro *and it lies in a cluster with another *Chlamydiaceae*-specific protein (CT712) of unknown function [51]. OmcA (CT444) is a well characterized chlamydial envelope-related protein (discussed in the previous section) that is found to be *Chlamydiaceae*-specific in our work. None of these proteins could be detected in the *Protochlamydia *by BLASTp searches using *Chl. trachomatis *proteins. Unlike the Pmps and Omps, chlamydial Inc proteins are localized to the chlamydial inclusion membrane. Different Inc proteins share minimal primary sequence identity with each other or with other proteins in the databases making the assignment of specific function difficult. Studies of individual Incs have shown that these peptides protrude from the surface of the inclusion and may mimic or bind to host cell proteins that selectively manicure the surface of the inclusion to avoid fusion with phagosomes or the host cell exocytic pathway [38,52,53]. Two Inc proteins, IncB (CT232) and IncC (CT233), were found to be unique to various *Chlamydiaceae *species. IncC is secreted by the type III secretion system and is expressed early during the initial stages of infection, and the gene for this protein lies adjacent to the *incB *loci in a cluster [37,54].

Another *Chlamydiaceae *specific protein, YprS (CT392), contains cysteine-rich complement-type repeats which are implicated in ligand-binding. Based on these repeat sequences, this protein bears some similarity to the insect vitellogenin and yolk protein receptors (VgR/YPR) family of proteins [55]. Many other *Chlamydiaceae *specific proteins of unknown functions are present in a number of different clusters including CT142-CT144, CT288/CT289, CT565-CT568, CT577-CT579, CT646/CT647, CT695/CT696 and CT846-CT849.

### *Chlamydia*- and *Chlamydophila*- specific proteins

The *Chlamydia *and *Chlamydophila *constitute two of the genera within the *Chlamydiaceae *family [1,2]. Thus, it is likely that unique proteins will be found exclusively in the species belonging to these two groups of bacteria. The genomes of two *Chlamydia *(*Chl. trachomatis *and *Chl. muridarum*) and three *Chlamydophila *species (viz. *Chlam. pneumoniae, Chlam. caviae *and *Chlam. abortus*) have been sequenced. In our work, we have identified twenty unique proteins for each of these two genera. A brief description of these results is presented below.

Of the twenty *Chlamydia-*specific ORFs identified in this work (Table 3), 16 were hypothetical. Those proteins of predicted function were Incs, specifically IncD, IncE, IncF and IncG (CT115-CT118). These 4 ORFs are transcribed from a single operon in *Chlamydia *species and when expressed, are not uniformly distributed [56]. Instead, these peptides appear to be concentrated at discrete sites in the inclusion membrane, primarily at sites of membrane-RB contact which can be visualized by both immunofluorescence and electron microscopy [56]. Unlike IncD-G localization, chlamydial MOMP appears to be depleted at the point of contact of RBs with the inclusion membrane but is found throughout the rest of the chlamydial outer membrane [56]. The asymmetric distribution of these proteins suggests that RBs exhibit polarity. The presence of such diversity of chlamydial polypeptides in the inclusion membrane lends support to the concept that chlamydiae actively control the interactions of the inclusion with the host cell to maintain a highly specialized environment favourable to chlamydial replication, in a lineage-specific manner. The other *Chlamydia*-specific proteins are of unknown function and some of these are arranged in 3 additional clusters, consisting of loci CT134/CT135, CT226-CT229 and CT357/CT358.

Of the 20 *Chlamydophila*-specific proteins identified in this work (Table 4), 9 are hypothetical while 11 have been minimally characterized previously. Among this group, four putative lipoproteins were identified, whose functions are not yet understood (CCA00246, CCA00575, CCA00578, CCA00738). Additionally 6 proteins have been described as putative membrane proteins in *Chlam. abortus *(CCA00222, CCA00261, CCA00621, CCA00360, CCA00361 and CCA00434), of which the last three are thought to be localized to the inner membrane [16]. Loci CC00360 and CC00361 are part of a cluster, which is the only cluster found among *Chlamydophila*-specific ORFs. This group also contained a serine-rich protein of unknown function, annotated as YwbM (CCA00062), which is absent from other chlamydiae as well as all other organisms.

### *Protochlamydia*-specific proteins

*Protochlamydia amoebophila *provides the only completed genome of a *Chlamydiales *species that belongs to a family other than the *Chlamydiaeceae*. The comparative genomics studies by Horn et al. [11] determined which of the *Protochlamydia *proteins were not present in other sequenced chlamydiae genomes. Although the list compiled by these authors contained over 1000 ORFs, which were present in *Protochlamydia *but were absent in other *Chlamydiales*, their study did not examine which of these proteins were also present in other bacterial phyla. We have undertaken this analysis in order to determine *Protochlamydia*-specific proteins. A total of 445 *Protochlamydial *ORFs were identified, and all were of unknown function. The listing of these proteins is provided in the Additional file 1. Most of these peptides were large in size (>300aa). Fifty-five gene clusters were found. These clusters were located at loci PC0055/56, PC0062-64, PC0074-76, PC0080/81, PC0117/18, PC0129/30, PC0293/94, PC0296/97, PC0302-04, PC0408-11, PC0463/64, PC0529/30, PC0535/36, PC0576-80, PC0611/12, PC0698/99, PC0726/27, PC0773/74, PC0812-14, PC0829/30, PC0833/34, PC0836-43, PC0857/58, PC0869/70, PC0885/86, PC0910/11, PC0967/68, PC0982/83, PC1051/52, PC1150-55, PC1204-08, PC1223/24, PC1233/34, PC1283/84, PC1294/95, PC1380/81, PC1387/88, PC1414/15, PC1417/18, PC1517/18, PC1531/32, PC1581-83, PC1621/22, PC1642/43, PC1685-87, PC1692-94, PC1741/42, PC1773-75, PC1789/90, PC1795/96, PC1826/27, PC1870/71, PC1891-94, PC1902-04, and PC1959-61.

### Cases of putative gene loss or transfer

The impact of gene acquisition and loss on bacterial genome evolution is currently a central question in microbiology which remains unresolved. It was therefore of interest to identify cases in which a gene appears to have been lost or possibly transferred from chlamydial genomes. Such cases were detected when all other species of a particular chlamydial group (e.g. *Chlamydiales*, *Chlamydiaceae *etc) contained a specific protein, with the exception of one or only a few species. In all, 33 such cases were identified among the different chlamydial groups (Table 5). Interestingly, 10 proteins which were found in the *Chlamydophila *and *Protochlamydia *were absent in the two available *Chlamydia *species (CCA00154, CCA00168, CCA00180, CCA00266, CCA00333, CCA00495, CCA00619, CCA00733, CCA00743, CCA00855). These genes may represent incidences of gene loss in the *Chlamydia*, or possible gene transfer between the *Chlamydophila *and *Protochlamydia*. Some phylogenies based on rRNA support the early separation of *Chlamydia *[57,58]. However, indel analysis and some protein phylogenies indicate that the *Chlamydophila *are ancestral to the *Chlamydia *[25]. Among the results, 6 proteins were found in all *Chlamydiaceae *species with the exception of *Chl. trachomatis *(CCA00231, CCA00396, CCA00425, CCA00538, CCA00827, CCA00910). Furthermore, 6 different proteins were found in all *Chlamydiaceae *species except for *Chlam. pneumoniae *(CT049, CT050, CT051, CT214, CT867, CT868). Also, 2 proteins were missing from *Chlam. caviae *which were found in all other available chlamydial species (CT244 and CT277), while 2 different proteins were absent in *Chl. muridarum *which were present in other *Chlamydiaceae *genomes (CCA00470, CCA00259). One protein which was unique to all *Chlamydiales *(CT550) and two proteins which were found in various *Chlamydiaceae *(CT444.1 and CT702) were also not found in the *Chlam. abortus *genome. One protein was found which was uniquely shared by all *Chlamydia *species and *Chlam. pneumoniae*, to the exclusion of all other *Chlamydophila *which may represent a lateral gene transfer (CCA00075). A single protein was absent in *Chlam. pneumoniae *which was present in all other *Chlamydophila *genomes (*Chlam. caviae *and *Chlam. abortus*) (CT785). Since *Chl. trachomatis *and *Chlam. pneumoniae *were used as probes, it is possible that other genes may have been lost from these genomes which were not identified during the searches. When the functions of these proteins are elucidated, these patterns of putative gene loss will likely shed light on their importance in regards to the differences in chlamydial biology, as well as why such gene losses may have occurred. Another well-studied example of a *Chlamydiales *protein that is also present in various *Rickettsiales *as well as various plants and algal plastids, consist of the nonmitochondrial ADP-ATP translocases (CT065 and CT495). Phylogenetic studies indicate that these genes have been transferred from a *Chlamydiales *ancestor to the other groups where these proteins are found [59,60].

### Temporal expression of Chlamydiae-specific genes

The developmental cycle of chlamydiae is regulated at the transcriptional level, although this regulation is not well understood [61]. Determining the stage-specificity of different genes is important for understanding the development and function of different chlamydial species. In vivo expression studies [62], in vivo transcription studies [47], and transcriptosome studies [63] have been carried out. The different lineage-specific proteins were compared with microarray studies carried out by Nicholson et al. [63]. Of all the group-specific proteins identified in this work, 61 chlamydiae loci were determined to be regulated in this manner, and fall into one of the following 4 categories as divided by Nicholson et al. [63]): early (6-24 hours post infection (hpi)), midlate I (12-24 hpi), midlate II (18-24 hpi) or late (24-36 hpi). The results for the stage-specific expression of these proteins are summarized in Table 6. IncC, a *Chlamydiaceae*-specific protein, is the only identified ORF in this study to be expressed at the early time point. Of the midlate I expressed genes, all the *Chlamydiales*-specific proteins were hypothetical (CT053, CT066, CT273, CT504, CT635, CT666, CT667, CT670, CT734). Of the midlate I *Chlamydiaceae*-specific proteins, LtuA (CT377) and PmpB (CT413), Pmp F-I (CT870-2, CT874) were the only ORFs of known function, while others (CT289, CT372, CT618, CT668, CT671) remain to be understood. Interestingly, only *Chlamydiaceae*-specific molecules and 2 putatively lost/transferred genes were found to be expressed during the midlate II phase. These *Chlamydiaceae*-specific proteins consisted of the clustered loci CT412/413, as well as OmpA (CT681) and OmpB (CT713). The lost/transferred genes, CT049 and CT051, were hypothetical proteins of unknown function. Many temporally expressed chlamydiae-specific genes were found in the late phase of the developmental cycle. Of the *Chlamydiales *proteins, OmcA (CT444) and OmcB (CT443) were the only proteins of known function while the remaining 4 were hypothetical (CT016, CT017, CT181 and CT546). Several interesting proteins were expressed late in the *Chlamydiaceae*-specific category including HctB (CT046), LtuB (CT080), YprS (CT392), SRP (CT442), the clustered loci OmcA (CT444) and OmcB (CT443), PmpE (CT869) and the clustered loci CT578/579 and CT082/083. All other differentially expressed proteins during this phase were hypothetical regardless of chlamydial lineage; *Chlamydiaceae *(CT005, CT288, CT365, CT552, CT565, CT620, CT695, CT712, CT848), *Chlamydia *(CT249, CT694) and lost/transferred genes (CT050, CT214, CT702, CT868).

### Divergence rates of Chlamydiae-specific proteins

We have also examined the sequence divergence rates for chlamydiae-specific proteins as compared to more ubiquitous proteins to determine whether the former proteins are evolving at a much faster rate. A relative measure of this was obtained by determining the degree of sequence conservation (as measured by % amino acid identity) among different chlamydial species for a number of *Chl. trachomatis *proteins that are either *Chlamydiales*-specific or which are more broadly distributed among bacteria. Results of these analysis for the two classes of proteins (i.e. *Chlamydiales*-specific vs broadly distributed) are presented in Tables 7A and 7B, respectively. As seen, in both cases, *Chl. trachomatis *proteins exhibited highest identity (~75–90%) to the *Chl. muridarum *homologs. This was followed by the species from the *Chlamydophila *genus (41–62% identity for the chlamydiae-specific proteins vs 60–80% identity for the broadly distributed group) and the *P. amoebophila*. The overall pattern that was observed was very similar for the two sets of proteins, although the proteins that are more broadly distributed were found to exhibit between 5–15% higher degree of conservation (i.e. slower rate of divergence) in comparison to the *Chlamydiales*-specific proteins.

## Conclusion

Chlamydial species are among the 'high profile' pathogens of the 21^st ^century [1]. These species are responsible for a vast array of serious diseases in humans and animals. Our understanding of the chlamydial diversity, host range, environmental distribution, as well as clinical involvement is presently limited and significantly underestimated. In this work, we have determined a large number of proteins which are uniquely present in various known chlamydiae species at different taxonomic levels. The whole protein signatures that we have identified include 59 ORFs that are unique to various known *Chlamydiales*, 79 ORFs unique for the *Chlamydiaceae *family, 20 unique ORFs each for the *Chlamydia *and *Chlamydophila *genera, as well as 445 ORFs that are presently distinctive of the *P. amoebophila *(Table 8) The smaller numbers of unique ORFs that are found in the *Chalmydiaceae *species in comparison to the *P. amoebophila *may be related to their smaller genome sizes and also the fact that former species grow in a more stable niche as compared to the latter species [11]. Also, 31 cases of putatively lost genes from the different chlamydial genomes were detected. The simplest and most parsimonious explanation for these shared unique signature proteins is that they were introduced only once in a common ancestor of the indicated chlamydial taxonomic groups located at different phylogenetic depths and then vertically passed on to species that descended from that particular ancestor [64,65]. In our analysis, we have also found some examples of chlamydiae-specific proteins which show sporadic species distribution (e.g. present in only a few *Chlamydia *and *Chlamydophila *species, but not in other species from these genera). Such proteins could possibly arise from two different mechanisms. First, it is possible that such genes were introduced initially at a deeper phylogenetic level (i.e. order or family) and then selectively lost in some species from the latter branching taxa. Alternatively, their distribution can be explained if the gene in question was initially introduced in one chlamydia species and then laterally transferred to some others, as is the case for the nonmitochondrial ADP-ATP translocases [59,60]. The numbers of such proteins is relatively small in comparison to the proteins that show taxa specific distribution.

**Figure 1:**
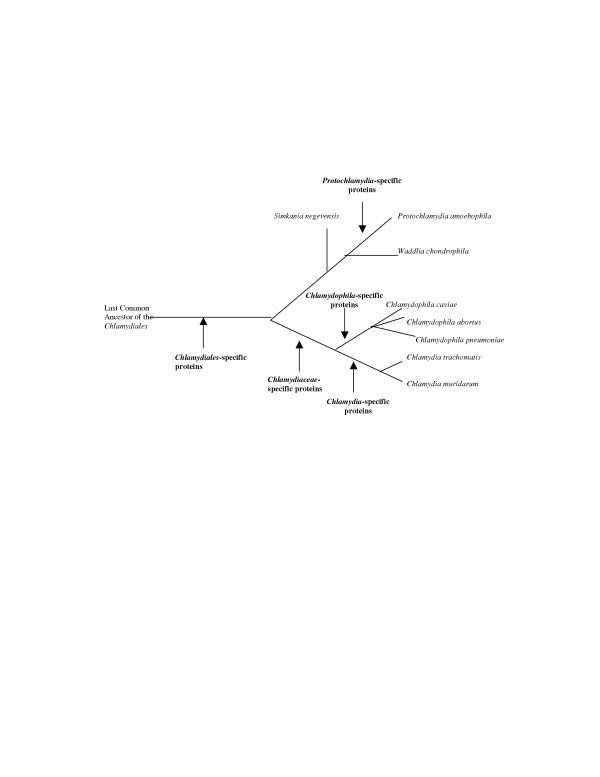
The branching pattern of the chlamydiae species based upon the distribution pattern of different chlamydiae specific proteins. The evolutionary stages where different proteins are indicated to have evolved are marked. The observed relationship is same as that seen in the phylogenetic trees based on 16S rRNA and protein sequences [1,2,25].

We have recently described a number of molecular signatures consisting of conserved indels (i.e. inserts and deletions) in widely distributed proteins viz. RNA polymerase α subunit, elongation factor-Tu, elongation factor-P, DNA gyrase B and lysyl-tRNA synthetase, that are distinctive characteristics of all chlamydial species [25]. The sequence information for these proteins was also obtained from *Simkania negevensis, Waddlia chondrophila*, and in a number of cases for *Neochlamydia hartmanellae*, covering all families within the *Chlamydiae *phylum. The unique presence of these conserved indels in all chlamydiae species provides strong evidence that they are distinctive characteristics of the entire *Chlamydiales *order. In phylogenetic trees based on a combined dataset of these protein sequence as well as 16S rRNA, the traditional *Chlamydiaceae *species (i.e. *Chlamydophila *and *Chlamydia*) and the chlamydiae-like organisms (viz. *Simkania*, *Waddlia *and *Parachlamydia*) were found to form two distinct clades indicating that these two groups have diverged from each other very early in the evolution of chlamydiae. The chlamydiae-specific proteins identified in the present work provide additional powerful means for understanding and constructing a reliable phylogeny of the *Chlamydiales*. Based upon the shared presence of these chlamydial-specific proteins, different taxonomic clades or groups within the *Chlamydiales *could be identified. Figure 1 shows a tree indicating branch points marked by the different chlamydiae group-specific proteins. In addition to distinguishing *P. amoebophila *from other *Chlamydiaceae *species, these proteins also support the distinctness of the two genera (i.e. *Chlamydophila *and *Chlamydia*) within this family. The phylogenetic inferences based on these proteins are in complete agreement with the relationships observed within this phylum based on different proteins as well as 16S rRNA trees [2,8,25]. The genes encoding this set of peptides were likely acquired or evolved sometime after the divergence of the *Protochlamydia *and the other chlamydia-like organisms. Although sequence information for other chlamydiae (viz. *Simkania *and *Waddlia*) is lacking at present, it is likely that many of the *Chlamydiales*-specific proteins identified here will also be found in these species. Further, the clustering of the chlamydiae-like species in phylogenetic trees [2,25]suggests that many of the proteins which are presently indicated to be specific for *P. amoebophila *may also be found in the other chlamydiae-like organisms and they may provide unique molecular markers for these groups of species.

The analyses of bacterial genomes have indicated that a substantial proportion of their gene repertoire is comprised of signature proteins or ORFans i.e., open reading frames which have no known homologs and consequently no known function [64-66]. The mechanisms by which such novel genes arise in different genomes are presently not clearly understood and they have been discussed in other recent publications [67-69]. However, it is possible that homologs of some of these genes/proteins are also present in other bacteria but because of their extensive sequence divergence significant similarity to the chlamydiae homologs is not observed. One example where this may be the case is that of the OmcA (CT444) protein, which is listed as a *Chlamydiaceae*-specific protein in this study (Table 2). An OmcA homolog is annotated in *P. amoebophila *genome because it is cysteine-rich and upstream of a large cysteine-rich protein, but it does not show significant similarity to the *Chlamydiaceae *homologs in BLASTp searches. Another protein that we have not included in any of the Tables but is of interest is CT020 (signal peptidase I). The homologs of this protein are highly conserved in all chlamydiae (E values ranging from 0 to 1e-101) and there is a large drop in E value from chlamydiae to the first non-chlamydiae hit (E value 3e-09), which is also indicated as signal peptidase I. The sequence of this protein has again greatly diverged between chlamydiae and non-chlamydiae species. Although the presence of these proteins could be explained by extensive sequence divergence, the possibility that they may have originated independently cannot be excluded. Our survey of the divergence rates of proteins that are either specific for the *Chlamydiales *vs those that are broadly distributed in different bacteria, indicate that *Chlamydiales*-specific proteins exhibit only slightly higher divergence rate (~10%) in comparison to the more broadly distributed proteins. This difference in our view is unlikely to be generally responsible for the failure to detect the orthologs of most of the chlamydial-specific proteins in other species.

Most of the chlamydiae-specific proteins that have been identified in the present work are of unknown function. Many of these genes are present in distinct clusters in genomes indicating that these clusters could comprise functional units and the corresponding proteins may be involved in related cellular functions [26,70]. The studies aimed at understanding the cellular functions of the *Chlamydiales*-specific proteins (i.e. their localization, expression and function) should prove highly informative for understanding chlamydial development, virulence and infectivity. The studies on the *Chlamydiaceae*-specific proteins should provide important insights regarding characteristics that distinguish them from other chlamydiae families. Additionally, a large number of protein coding sequences that are specific for either particular genera or different chlamydial species have been identified. The functional studies of such proteins could provide important information regarding factors, which are presumably responsible for their different properties such as tissue tropism and disease spectrum.

**Figure 2:**
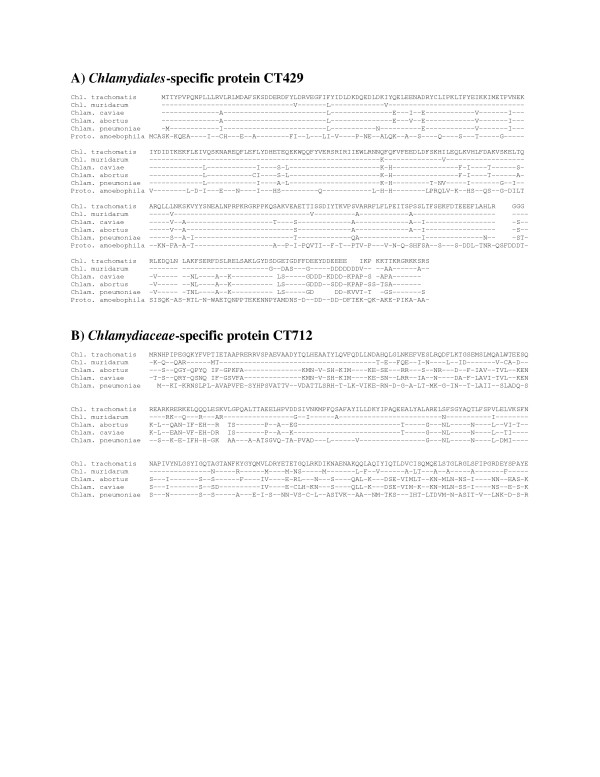
Multiple sequence alignments of a representative *Chlamydiales*-specific protein CT429 (A), and a *Chlamydiaceae*-specific protein CT712 (B). Both of these proteins are of unknown function and they are uniquely found in the indicated groups of species. The sequence alignments for all available homologs for these proteins are shown. Dashes (-) in the alignments indicate identity with the amino acid on the top line (i.e. *Chl. trachomatis *sequence). Both these proteins contain many highly conserved regions suitable for PCR primer design that can be used for diagnostic purposes.

The identified chlamydiae-specific proteins also provide novel biomarkers that should prove of much value in the diagnosis of these bacteria and in exploration of their prevalence and diversity. The identification and screening of chlamydiae and chlamydiae-like organisms in the past has relied on a small number of biomarkers (e.g. 16S and 23S rRNA, MOMP, GroEL protein, lipopolysaccharide epitope Kdo, small cysteine-rich lipoprotein and 60-kDa cysteine-rich protein) [71-74]. In this context, our identification of numerous proteins, which are uniquely found in either various *Chlamydiales, Chlamydiaceae, Chlamydophila *or *Chlamydia *groups of species is of great interest and they provide novel and valuable means for detection of these groups of species. Many of these group-specific proteins are highly conserved and sequence alignment for one *Chlamydiales*-specific protein (CT429) and one *Chlamydiaceae*-specific protein (CT712) are presented in Figure 2. The sequence alignments of both these proteins contain many highly conserved regions that are suitable for design of PCR primers that should enable successful amplification of all species belonging to these groups. Monoclonal and polyclonal antibodies based upon different chlamydial-specific proteins provide another means for diagnostic studies. Thus, based upon different group-specific proteins that have been identified in the present work, it should be possible to develop novel diagnostics that are capable of distinguishing different groups of chlamydiae species (viz. *Chlamydia*, *Chlamydophila*, *Chlamydiaceae *and *Chlamydiales*) from each other with high degree of accuracy.

## Methods

### Identification of Chlamydiae-specific proteins

In order to identify proteins which are chlamydiae-specific, systematic BLAST searches were carried out on different proteins in the genomes of *Chl. trachomatis *and *Chlam. caviae *against all available sequences in the databases. BLASTp searches were performed using each of the proteins or ORFs in these genomes as probes to identify all other bacteria which contain related protein sequences [75]. The searches were carried out using default search parameters, as set by the protein-protein BLAST program, which included the low complexity filter. The low complexity filter removes regions of query sequence which are highly repetitive and which could lead to spurious high similarity with unrelated proteins. The results obtained were visually inspected for homologs showing specificity to the chlamydiae with no other similar homologs present in any other bacteria. This was done by analyzing expect values (E values) for all proteins. The Expect value (E value) represents the statistical significance of a hit in a BLAST search. This number equates to the number of hits that one would find by chance when searching a database of a particular size. The E values depend upon the length of the protein as well as the extent of sequence homology that is observed with any given protein. The E values which are very low (generally less than e-200) are indicated in the results obtained as '0'. In our extensive work, the E value for the top hit (i.e. to the query itself) is generally found to be '0', when the query protein is of a length longer than 325 aa. For proteins of smaller lengths, the E value for the top hit has a finite value. The E value of top hits may be slightly affected by the application of the filter which effectively shortens the query length [75]. Proteins were considered *Chlamydiales*-specific if all hits belonged to the chlamydiae species, or if the next best non-chlamydiae hit had an expect value which was not significant and could occur by chance. BLAST results with E values > 10^-4 ^are in range where the observed similarity could occur by chance. All such hits were examined for protein lengths to ensure that it was similar to that of the query protein before it was considered as a related protein. Occasionally higher E-values were permitted in analysis when the length of the query protein was small as with fewer characters the E values are higher and this often produce hits with higher E-values (i.e. > 10^-4^) that are significant for the study. It should be mentioned that BLAST searches can sometime indicate misleading relationships, particularly when no close relatives of the query species are in the database [76]. However, in the present study where most of the reported BLAST hits are for chlamydiae species, for which there are several published genome, such a possibility is highly unlikely. For all chlamydiae-specific proteins, E-values for each chlamydiae BLAST hit, as well as the first non-chlamydial hit are reported here. Furthermore, the sizes of each protein in *Chl. trachomatis *(or other genomes that were used as BLAST probes) are also listed, and the sizes of other chlamydial homologs are highly similar, unless it is otherwise stated. The same criteria were used for the detection of *Chlamydiaceae*-specific, *Chlamydia*-specific and *Chlamydophila*-specific proteins. For clarification purposes, the chlamydiae loci and accession numbers are given as reference for the different proteins. The genome of *P. amoebophila *was also examined in this manner to identify proteins that are specific for *Protochlamydia*. Those genes which were found in all species within a given group (i.e. *Chlamydiales*, *Chlamydiaceae *etc) except for one or a few species, were considered to be due to gene loss or lateral gene transfer.

### Alignments of Chlamydiae-specific proteins and percentage identity determination

The global alignments of *Chlamydiales*-specific protein CT429 and *Chlamydiaceae*-specific protein CT712 were carried out by first retrieving chlamydiae sequences from the NCBI database [77] in FASTA format. ClustalX program [78] was then used to construct both alignments using the default parameters. Pair-wise alignments of sequences for determining percentage identity between *Chl. trachomatis *proteins and those from other chlamydial species were carried out using the ALIGN Plus 4 program package (Scientific and Educational Software) using the BLOSUM 62 scoring matrix [79] and the default parameters of the alignment program.

### Analysis of temporal expression of Chlamydiae-specific proteins

Lineage-specific chlamydial proteins were compared to the microarray data of Nicholson et al. [63]. In that work, the expression patterns of *Chl. trachomatis *genes were divided into different stages: early (6–24 hours post infection), midlate I (12–24 hpi), midlate II (18–24 hpi) or late (24–36 hpi). Proteins identified in that work which exhibited at least 3-fold differences in expression patterns at different stages, were then placed into one of these different groups. The information for stage-specific expression of chlamydiae-specific proteins was extracted from this study.

## Authors' contributions

MSV was assigned to carry out the initial BLAST searches on *Chl. trachomatis *and *Chlam. pneumoniae *genomes to identify chlamydial specific proteins. Subsequently, EG was asked to confirm these results and she also carried out BLAST searches on *Protochlamydia *proteins to identify proteins that are unique to this species. EG also prepared an initial draft of the manuscript. RSG was responsible for conceiving and directing this study from the beginning to the end, for the final evaluation of all results, and for preparing the final submitted manuscript. All authors have read and approved the final manuscript.

## Figures and Tables

**Table 1:** List of the *Chlamydiales*-specific proteins

Protein Loci	Protein (Name/Size)	C. tra	C. mur	C. pne	C. cav	C. abo	Protochlam.	Next best BLAST Hit (E value, protein length)
CT007 (AAC67597)	hypoth, 316aa	0	8e-164	8e-108	6e-108	3e-105	8e-10, 320aa	*Plasmodium yoelii *(0.063, 1491aa)
CT011 (AAC67601)	hypoth, 418aa	0	0	7e-151	2e-155	1e-157	7e-47, 445aa	*Desulfotalea psychrophila *(1e-06, 337aa)
CT016 (AAC67606)	hypoth, 242aa	2e-139	3e-119	1e-82	4e-89	3e-86	2e-37, 233aa	*Rickettsia sibirica *(0.20, 1851aa)
CT017 (AAC67607)	hypoth. 433aa	0	0	1e-122	7e-133	9e-135	4e-52, 431aa	*Thermo. tengcongensis *(1e-06, 306 aa)
CT021 (AAC67611)	hypoth, 247aa	2e-139	4e-107	1e-47	3e-56	4e-53	2e-13, 245aa	*Xenopus laevis *(1.7, 600aa)
CT031 (AAC67621)	hypoth, 100aa	4e-51	4e-42	2e-25	4e-26	2e-26	0.010, 107aa	*Mus musculus *(3.6, 384aa)
CT038 (AAC67628)	hypoth, 116aa	1e-63	2e-49	4e-25	1e-25	2e-23	2e-09, 109aa	*Neurospora crassa *(2.8, 1223aa)
CT053 (AAC67644)	hypoth, 148aa	2e-61	4e-57	2e-51	1e-50	1e-48	5e-29, 155aa	*Xenopus tropicalis *(4.7, 1049aa)
CT066 (AAC67657)	hypoth, 158aa	3e-77	1e-75	8e-65	3e-65	5e-65	6e-41, 165aa	*Vibrio parahaemolyticus *(1.8, 92aa)
CT079 (AAC67670)	hypoth, 147aa	4e-81	1e-71	2e-06	1e-29	1e-29	3e-06, 172aa	*Leuconostoc mesenteroides *(0.11, 263aa)
CT131 (AAC67722)	PTMP, 1138aa	0	0	0	0	0	3e-33, 1348aa	*Plasmodium yoelii *(0.31, 592aa)
CT181 (AAC67772)	hypoth, 236aa	7e-131	1e-113	3e-77	7e-81	4e-80	2e-10, 200aa	*Acinetobacter sp*. (0.94, 371aa)
CT260 (AAC67853)	hypoth, 163aa	9e-91	9e-81	5e-62	3e-68	1e-67	4e-22, 159aa	*Sinorhizobium meliloti *(0.18, 166aa)
CT266 (AAC67859)	hypoth, 393aa	0	5e-179	9e-103	5e-120	2e-119	4e-08, 498aa	*Xanthomonas axonopodis *(2.8, 189aa)
CT271 (AAC67864)	hypoth, 95aa	1e-39	2e-36	7e-25	1e-25	9e-26	1e-10, 97aa	*Gibberella zeae *(3.6, 1362)
CT273 (AAC67866)	hypoth, 188aa	2e-89	1e-77	2e-60	5e-63	2e-63	0.001, 149aa	*Corynebacterium efficiens *(0.34, 640aa)
CT274 (AAC67867)	type III secretion chaperone, 139aa	3e-74	3e-70	1e-61	1e-62	1e-61	3e-31, 131aa	*Synechococcus elongatus *(2e-05, 363aa)
CT303 (AAC67896)	hypoth, 171aa	2e-81	5e-66	2e-40	2e-47	6e-47	3e-04, 177aa	*Yersinia pestis *(1.3, 373aa)
CT309 (AAC67902)	hypoth, 266aa	2e-144	2e-129	8e-86	1e-93	5e-94	2e-29, 263aa	*Methanococcus jannaschii *(2.6, 434aa)
CT312 (AAC67905)	pred. ferredoxin, 123aa	6e-67	6e-54	1e-30	7e-36	1e-35	1e-04, 130aa	*Pan troglodytes *(1.2, 1524aa)
CT350 (AAC67945)	hypoth, 566aa	0	0	2e-141	3e-157	1e-157	2e-30, 561aa	*Nostoc sp*. PCC 7120 (7e-06, 1547aa)
CT351 (AAC67946)	hypoth, 697aa	0	0	0	0	0	5e-53, 720aa	*Rattus norvegicus *(3.4, 389aa)
CT355 (AAC67950)	hypoth, 353aa	0	3e-161	3e-101	8e-101	1e-102	1e-21, 375aa	*Chromobacterium violaceum *(0.82, 556aa)
CT384 (AAC67980)	hypoth, 539aa	0	0	9e-106	4e-113	1e-111	2e-14, 580aa	*Pseudomonas fluorescens *(0.29, 314aa)
CT387 (AAC67984)	hypoth, 691aa	0	0	0	0	0	4e-160, 740aa	*Rubrobacter xylanophilus *(5e-10, 263aa)
CT421.2 (AAC68031)	hypoth, 53aa	5e-23	2e-22	5e-19	7e-20	4e-19	2e-12, 52aa	no other BLAST hits
CT425 (AAC68022)	hypoth, 621aa	0	0	0	0	0	0, 665aa	*Bordetella parapertussis *(4e-10, 767aa)
CT429 (AAC68026)	hypoth, 329aa	4e-165	4e-162	5e-149	2e-151	7e-151	8e-96, 388aa	*Geobacter sulfurreducens *(2e-04, 146aa)
CT443 (AAC68042)	OmcB, 553aa	0	0	0	0	0	4e-66, 558aa	*Rhodopirellula baltica (3e-25, 907aa)*
CT446 (AAC68045)	EUO protein, 183aa	1e-78	8e-72	1e-50	2e-53	1e-52	4e-41, 139aa	*Magnaporthe grisea *(2.1, 1259aa)
CT474 (AAC68074)	LysM protein, 336aa	2e-168	9e-116	5e-59	9e-71	7e-71	3e-04, 482aa	no other BLAST hits
CT504 (AAC68105)	hypoth, 288aa	2e-127	1e-105	4e-64	2e-69	2e-68	1e-23, 376aa	*Mus musculus *(3.0, 961aa)
CT538 (AAC68140)	hypoth, 238aa	8e-124	1e-120	5e-105	1e-108	1e-108	9e-65, 244aa	*Fritschea bemisiae *(3e-59, 241aa) *Enterococcus faecalis *(0.067, 541aa)
CT546 (AAC68148)	predicted OMP, 289aa	3e-163	4e-157	6e-87	1e-92	1e-93	3e-23, 249aa	*Legionella pneumophila *(0.042, 254aa)
CT547 (AAC68149)	hypoth, 318aa	2e-175	7e-158	8e-90	1e-92	2e-93	8e-29, 468aa	*Caenorhabditis elegans *(4e-04, 1168aa)
CT548 (AAC68150)	hypoth, 194aa	3e-89	2e-67	1e-48	2e-49	3e-48	0.004, 185aa	*Drosophila melanogaster *(0.17, 826aa)
CT556 (AAC68158)	hypoth, 159aa	1e-92	1e-82	9e-59	1e-60	1e-59	2e-06, 157aa	*Xenopus laevis *(1.8, 1636aa)
CT560 (AAC68162)	hypoth, 278aa	1e-135	2e-125	1e-86	4e-94	5e-93	0.002, 281aa	no other BLAST hits
CT573 (AAC68175)	hypoth, 409aa	0	1e-179	9e-90	3e-113	2e-106	1e-43, 472aa	*Pseudomonas syringae *(0.46, 9376aa)
CT583 (AAC68185)	Virulence plasmid pGP6 related protein, 263aa	5e-134	6e-118	4e-82	4e-91	2e-92	1e-46, 245aa	*Crocosphaera watsonii *(0.036, 333aa)
CT590 (AAC68192)	hypoth, 954aa	0	0	0	0	0	8e-96, 989aa	*Porphyromonas gingivalis *(0.003, 645aa)
CT602 (AAC68205)	hypoth, 130aa	5e-60	1e-55	2e-35	3e-37	1e-37	2e-19, 131aa	*Actinobacillus pleuropneumoniae *(2.8, 300aa)
CT616 (AAC68220)	hypoth, 429aa	0	0	2e-101	8e-115	3e-114	2e-09, 435aa	*Giardia lamblia *(0.003, 1024aa)
CT623 (AAC68227)	76 kDa Homolog, 446aa	0	2e-155	5e-152	1e-154	1e-157	1e-28, 470aa	*Mycobacterium tuberculosis *(0.18, 550aa)
CT631 (AAC68235)	hypoth, 84aa	1e-32	2e-31	1e-24	3e-28	8e-28	1e-11, 82aa	No other BLAST hits
CT635 (AAC68239)	hypoth, 144aa	2e-62	5e-58	6e-45	1e-50	2e-51	4e-04, 146aa	*Gallus gallus *(0.003, 1748aa)
CT642 (AAC68246)	hypoth, 271aa	2e-134	2e-116	2e-74	2e-79	8e-81	2e-22, 300aa	*Rattus norvegicus *(1.6, 509aa)
CT652.1 (AAC68830)	hypoth, 59aa	5e-26	3e-24	7e-12	3e-12	7e-13	3e-05, 75aa	*Parachlamydia sp*. (0.12, 70aa)
CT666 (AAC68261)	hypoth, 83aa	5e-40	9e-31	3e-35	1e-36	9e-37	0.43, 92aa	*Sacchromyces cerevisiae *(3.7, 604aa)
CT667 (AAC68262)	hypoth, 149aa	3e-78	1e-65	3e-51	9e-55	4e-55	8e-16, 154aa	*Legionella pneumophila *(0.005, 577aa)
CT670 (AAC68265)	hypoth, 168aa	1e-29	1e-27	2e-23	4e-24	3e-23	9e-10, 165aa	No other BLAST hits
CT734 (AAC68329)	hypoth, 221aa	7e-106	1e-95	2e-51	1e-56	2e-57	1e-08, 240aa	*Caenorhabditis briggsae *(1.1, 1072aa)
CT764 (AAC68359)	hypoth, 268aa	2e-154	4e-113	1e-46	7e-59	4e-59	2e-05, 256aa	*Tetraodon nigroviridis *(1.5, 388aa)
CT788 (AAC68383)	leader (60) peptide-periplasmic, 166aa	1e-79	3e-61	9e-25	4e-34	1e-32	0.003, 158aa	*Caenorhabditis elegans *(0.19, 2271aa)
CT809 (AAC68405)	hypoth, 103aa	1e-55	2e-44, 146aa	5e-17	2e-23	2e-21	4e-05, 158aa	*Canis familiaris *(2.1, 1831aa)
CT838 (AAC68435)	YjgP/YjgQ family protein, 366aa	0	0	e-126	e-130	3e-128	6e-24, 360aa	*Prochlorococcus marinus *(0.18, 592aa)
CT845 (AAC68442)	hypoth, 92aa	2e-46	7e-33	1e-19	2e-17	1e-17	8e-10, 85aa	*Candida albicans *(2.7, 557aa)
CT849.1 (AAC68455)	hypoth, 62aa	2e-28	9e-24	5e-10	2e-09	1e-08	1e-05, 81aa	No other BLAST hits
CT865 (AAC68463)	hypoth, 329aa	2e-180	8e-146	7e-76	1e-77	4e-78	9e-23, 352aa	*Bacteroides thetaiotaomicron *(0.20, 808aa)

The *Chlamydiales*-specific proteins were identified by systematic BLASTP searches using *Chl. trachomatis *ORFs as probes, where the only significant BLAST hits obtained were for chlamydiae species (see Methods section). Accession numbers of the query sequences are shown beside the *Chl. trachomatis *loci (CT number) in the first column. The observed expected (E) values for various chlamydiae homologs as well as the first non-chlamydial hit in the BLAST results are given. E values of 0.0 indicate an extremely high degree of similarity between proteins sequences. The second column describes the function and the size of each query protein (protein sizes are described for other chlamydiae only if they noticeably differ from the query protein). Abbreviations are: C. tra, *Chl. trachomatis*; C. mur; *Chl. muridarum*; C. pne, *Chlam. pneumoniae*; C. cav, *Chlam. caviae*; C. abo, *Chlam. abortus*; hypoth, hypothetical protein; Omc; Outer membrane cytochrome; OMP, outer membrane protein; Protochlam, *Protochlamydia*; Pred., Predicted; PTMP, Possible transmembrane protein.

**Table 2:** A Listing of the Identified *Chlamydiaceae*-specific proteins

Protein Loci	Protein (Name/Size)	C. tra	C. mur	C. pne	C. cav	C. abo	Next best BLAST hit (E value, protein length)
CT006 (AAC67596)	hypoth, 189aa	3e-95	6e-72	3e-12	9e-10	2e-09	*Listeria anguillarum *(0.45, 314aa)
CT005 (AAC67595)	hypoth, 363aa	1e-117	4e-63	3e-18, 417aa	8e-20	1e-20	*Ashbya gossypii *(0.86, 2272aa)
CT018 (AAC 67608)	hypoth, 157aa	3e-89	5e-53	2e-10	8e-09	3e-06	*Acetabularia acetabulum *(3.9, 613aa)
CT046 (AAC67637)	Histone-like protein HC2, 203aa	2e-29	2e-25	2e-05, 172aa	8e-08, 152aa	2e-08	No other BLAST hits
CT080 (AAC67671)	LtuB protein, 97aa	3e-50	4e-37	3e-11	2e-13	9e-16	*Clostridium tetani *(0.73, 311aa)
CT082 (AAC67673)	hypoth, 560aa	0	0	7e-91	8e-103	2e-102	*Saccharomycecs cerevisiae *(2.0, 1537aa)
CT083 (AAC67674)	hypoth, 160aa	1e-71	1e-57	7e-30	4e-29	9e-25	*Pseudomonas putida *(0.17, 1162aa)
CT109 (AAC67700)	hypoth, 262aa	2e-149	9e-133	8e-66	4e-70	7e-70	*Plasmodium falciparum *(1.1, 1057aa)
CT142 (AAC67733)	hypoth, 285aa	3e-155	2e-109	1e-39	9e-50	6e-49	*Heterobasidion annosum *(5.0, 507aa)
CT143 (AAC67734)	hypoth, 280aa	3e-165	3e-123	2e-36	9e-55	4e-56	porcine (0.26, 725aa)
CT144 (AAC67735)	hypoth, 285aa	2e-143	3e-101	7e-28	9e-34	6e-31	*Staphylococcus aureus *(0.041, 154aa)
CT147 (AAC67738)	hypoth, 1449aa	0	0	3e-71	4e-88	4e-91	*Homo sapiens *(8e-10, 2442aa)
CT178 (AAC67769)	hypoth, 404aa	0	0	3e-114	3e-120	4e-122	*Thiobacillus denitrificans *(2e-15, 830aa)
CT195 (AAC67787)	hypoth, 363aa	0	2e-147	8e-55	4e-71	1e-68	*Escherichia coli *(1.5, 346aa)
CT232 (AAC67824)	IncB, 115aa	7e-45	1e-29	4e-11	2e-08	7e-10	*Canis familiaris *(0.59, 472aa)
CT233 (AAC67825)	IncC, 178aa	9e-75	3e-38	0.52, 203aa	4e-04	0.006,184aa	*Tetraodon nigroviridis *(0.5, 1010aa)
CT253 (AAC67846)	hypoth, 215aa	5e-108	2e-99	2e-67	5e-72	8e-73	*Schizosaccharomyces pombe *(0.12, 880aa)
CT263 (AAC67856)	hypoth, 196aa	7e-109	1e-72	5e-23	2e-31	1e-29	*Mycobacterium bovis *(2.4, 744aa)
CT288 (AAC67881)	hypoth, 563aa	0	3e-175	6e-16	5e-23	1e-25	*Debaryomyces hansenii *(4e-04, 2042aa)
CT289 (AAC67882)	hypoth, 379aa	3e-167	3e-95	6e-27	6e-38	5e-31	*Streptococcus pyogenes *(0.049, 535aa)
CT324 (AAC67917)	hypoth, 303aa	1e-161	8e-115	1e-50	6e-55	1e-55	*Brucella melitensis *(0.29, 263aa)
CT330 (AAC67925)	hypoth, 90aa	2e-44	1e-32	3e-07	3e-04	1e-05	*Oryza sativa *(2.7, 677aa)
CT338 (AAC67933)	hypoth, 153aa	1e-86	1e-66	1e-23	2e-29	5e-28	*Acinetobacter sp*. (1.6, 304aa)
CT365 (AAC67961)	hypoth, 575aa	0	4e-156	3e-58	7e-63	1e-61	No other BLAST hits
CT372 (AAC67968)	hypoth, 442aa	0	0	1e-114	2e-116	3e-119	*Bradyrhizobium japonicum *(0.016, 675aa)
CT377 (AAC67973)	LtuA, 46aa	4e-20	8e-19	2e-08	6e-11	5e-10	No other BLAST hits
CT383 (AAC67979)	hypoth, 243aa	7e-140	1e-88	3e-26	2e-35	2e-28	*Burkholderia fungorum *(2.9, 780aa)
CT392 (AAC67989)	yprS, 377aa	0	2e-116	1e-07	1e-14	3e-07	*Saccharomyces cerevisiae *(0.048, 1038aa)
CT412 (AAC68009)	PmpA, 975aa	0	0	8e-141	3e-179	4e-175	many other chlamydiae hits in between *Chloroflexus aurantiacus *(3e-05, 767aa)
CT413 (AAC68010)	PmpB, 1751aa	0	0	0	0	0	*Chloroflexus aurantiacus*, 8e-10, 767aa
CT421.1 (AAC68030)	hypoth, 53aa	7e-23	3e-20	4e-15	6e-12	1e-14	*Bradyrhizobium japonicum *(3.8, 803aa)
CT440 (AAC68039)	hypoth, 112aa	1e-58	2e-45	6e-08	1e-04	4e-07	*Homo sapiens *(0.017, 100aa) (partial)
CT442 (AAC68041)	15kDa SRP, 150aa	7e-70	1e-33	1e-04	1e-12	3e-12	No other BLAST hits
CT444 (AAC68043)	OmcA, 88aa	1e-39	4e-31	2e-12	6e-15	6e-15	Ovis aries (0.14, 197aa) **
CT466 (AAC68066)	hypoth, 109aa	5e-48	7e-39	2e-24	1e-26	1e-27	*Geobacter metallireducens *(0.55, 557aa)
CT471 (AAC68071)	hypoth, 200aa	5e-112	1e-103	1e-46	1e-62	5e-63	*Azoarcus sp*. (1.9, 168aa)
CT482 (AAC68082)	hypoth, 217aa	1e-115	1e-91	1e-49	6e-60	4e-64	*Legionella pneumophila *(1.8, 438aa)
CT483 (AAC68083)	hypoth, 121aa	1e-51	1e-36	6e-06	2e-08	4e-08	*Rubrobacter xylanophilus *(0.14, 293aa)
CT529 (AAC68131)	hypoth, 298aa	1e-151	2e-91	4e-36	1e-38	8e-42	*Aspergillus nidulans *(1.9, 274aa)
CT552 (AAC68154)	hypoth, 135aa	6e-72	1e-41	5e-04	3e-06	7e-07	*Guillardia theta *(0.19, 263aa)
CT565 (AAC68167)	hypoth, 147aa	7e-79	1e-69	1e-41	3e-46	7e-48	*Caenorhabditis elegans *(0.11, 317aa)
CT566 (AAC68168)	hypoth, 330aa	8e-175	6e-141	8e-86	4e-89	1e-87	*Vibrio vulnificus *(0.26, 506aa)
CT567 (AAC68169)	hypoth, 174aa	1e-87	9e-61	5e-24	2e-17	2e-17	*Takifugu rubripes *(4.1, 1374aa)
CT568 (AAC68170)	hypoth, 151aa	2e-71	4e-48	2e-22	2e-18	5e-18	*Giardia lamblia *(1.6, 6739aa)
CT577 (AAC68179)	hypoth, 119aa	1e-60	3e-48	6e-16	3e-26	2e-26	*Giardia lamblia *(0.050, 551aa)
CT578 (AAC68180)	hypoth, 487aa	8e-175	3e-150	4e-81	2e-99	1e-103	*Candida glabrata *(0.023, 592aa)
CT579 (AAC68181)	hypoth, 439aa	3e-141	8e-115	2e-76	4e-77	1e-81	No other BLAST hits
CT589 (AAC68191)	hypoth, 602aa	0	0	1e-126	1e-145	3e-146	*Bacillus subtilis *(0.052, 605aa)
CT618 (AAC68222)	hypoth, 266aa	1e-142	6e-87	8e-10	4e-19, 306aa	5e-22	*Drosophila melanogaster *(0.11, 1868aa)
CT620 (AAC68224)	hypoth, 838aa	0	0	0	1e-180	2e-177	*Borrelia burgdorferi *(0.29, 735aa)
CT646 (AAC68823)	hypoth, 459aa	0	0	3e-60	1e-68	5e-66	*Aspergillus nidulans *(5.9, 629aa)
CT647 (AAC68824)	hypoth, 192aa	2e-98	5e-65	2e-18	1e-15	3e-13	*Ciona intestinalis *(1.8, 407aa)
CT651 (AAC68828)	hypoth, 608aa	0	0	1e-97	7e-97	3e-95	*Bacteroides thetaiotaomicron *(0.15, 1003aa)
CT654 (AAC68249)	hypoth, 162aa	5e-81	9e-75	1e-27	1e-32	1e-33	*Mesocricetus auratus *(2.5, 210aa)
CT668 (AAC68263)	hypoth, 223aa	1e-114	9e-93	6e-69	5e-78	1e-76	*Bacillus halodurans *(0.045, 324aa)
CT671 (AAC68266)	hypoth, 283aa	2e-149	1e-115	2e-63	2e-76	1e-72	*Giberella zeae *(0.053, 595aa)
CT681 (AAC68276)	MOMP, 393aa	0	7e-176	2e-139	3e-137	2e-136	No other BLAST hits except chlamydiae
CT695 (AAC68290)	hypoth, 398aa	0	7e-147	6e-12	2e-23	2e-22	*Ustilago maydis *(0.44, 417aa)
CT696 (AAC68291)	hypoth, 403aa	0	3e-179	6e-59	3e-70	2e-73	*Mus musculus *(0.031, 708aa)
CT712 (AAC68307)	hypoth, 390aa	0	0	8e-101	1e-129	1e-132	*Mus musculus *(0.086, 3159aa)
CT713 (AAC68308)	PorB, 340aa	0	0	5e-120	2e-133	5e-132	No other BLAST hits except chlamydiae
CT718 (AAC68313)	hypoth, 174aa	3e-93	8e-81	3e-44	2e-46	6e-47	*Prochlorococcus marinus *(0.82, 250aa)
CT744 (AAC68339)	phosphoprotein, 821aa	0	0	2e-138	1e-153	3e-160	*Cytophaga hutchinsonii *(0.38, 466aa)
CT753 (AAC68348)	hypoth, 74aa	3e-34	4e-27	1e-13, 76aa	2e-13	2e-13	*Cycas *necrotic stunt virus (1.3, 559)
CT763 (AAC68358)	hypoth, 139aa	6e-72	3e-52	2e-28	3e-23	2e-22	*Drosophila pseudoobscura *(2.6, 773aa)
CT795 (AAC68390)	hypoth, 163aa	1e-79	7e-46	1e-13	3e-13	2e-14	*Pan troglodytes *(0.52, 924aa)
CT812 (AAC68408)	OmpD, 1531aa	0	0	0	0	0	*Rhodopirelulla baltica *(2e-04, 703aa)
CT846 (AAC68443)	hypoth, 234aa	1e-123	1e-108	7e-67	3e-64	1e-66	*Streptomyces coelicolor *(2.1, 689aa)
CT847 (AAC68444)	hypoth, 172aa	2e-88	8e-73	1e-53	1e-53	2e-52	*Saccharomyces cerevisiae *(0.001, 1434aa)
CT848 (AAC68445)	hypoth, 168aa	1e-76	5e-67	2e-55	6e-54	8e-54	*Drosophila pseudoobscura *(0.34, 1000aa)
CT849 (AAC68446)	hypoth, 159aa	4e-74	1e-64	2e-57	1e-56	4e-57	*Gallus gallus *(0.034, 1546aa)
CT860 (AAC68458)	hypoth, 493aa	0	1e-132	2e-56	4e-73	4e-77	*Leishmania major *(0.26, 1346aa)
CT861 (AAC68459)	hypoth, 506aa	0	7e-172	6e-67	9e-74	5e-76	*Vibrio fischeri *(0.008, 1023aa)
CT863 (AAC68461)	hypoth, 482aa	0	2e-150	7e-82	2e-86	7e-90	*Mus musculus *(0.006, 1043aa)
CT869 (AAC68467)	OmpE, 964aa	0	0	9e-108	1e-119	8e-112	*Escherichia coli *(9e-05, 1250aa)
CT870 (AAC68468)	OmpF, 1034aa	0	0	1e-40, 931aa	2e-41	5e-46	long list of chlamydiae homologs ; *Dictyostelium discoideum *(4e-04, 485aa)
CT871 (AAC68469)	OmpG, 1013aa	0	0	4e-127	7e-180	2e-175	long list of chlamydiae homologs ; *Desulfotalea psychrophila *(8e-07, 2363aa)
CT872 (AAC68470)	OmpH, 1016aa	0	0	6e-152, 978aa	0	0	long list of chlamydiae homologs ; *Crocosphaera watsonii *(0.28, 822aa)
CT874 (AAC68472)	OmpI, 878aa	0	0	2e-81, 930aa	9e-73, 942aa	2e-77	long list of chlamydiae homologs ; *Nostoc punctiforme *(0.006, 1851aa)

These proteins are uniquely found in various *Chlamydiaceae *species but not detected in *Protochlamydia *or other bacterial groups. Other details as in Table 1. **OmcA is an annotated ORF in the *P. amoebophila *genome, however it could not be detected using *Chl. trachomatis *as probe via BLASTp and so was not considered a *Chlamydiales *protein; SRP, sulfur rich protein.

**Table 3:** *Chlamydia*-specific proteins

Protein Loci	Protein Name/Size	C. tra	C. mur	Next Best Hit (E value, protein length)
CT115 (AAC67706)	IncD, 141aa	4e-45	5e-11, 124aa	No other BLAST hits
CT116 (AAC67707)	IncE, 132aa	2e-45	4e-14, 143aa	No other BLAST hits
CT117 (AAC67708)	IncF, 104aa	3e-29	6e-25, 104aa	No other BLAST hits
CT118 (AAC67709)	IncG, 167aa	5e-59	8e-11, 166aa	*Mesorhizobium loti *(8.2, 101aa)
CT135 (AAC67726)	hypoth, 360aa	4e-179	4e-115, 365aa	Hepatitis C virus (0.86, 414aa)
CT163 (AAC67754)	hypoth, 548aa	0	8e-13, 246aa	*Canis familiaris *(1.5, 1971aa)
CT174 (AAC67765)	hypoth, 151aa	9e-81	4e-10, 120aa	*Streptococcus pyogenes *(0.066, 152aa)
CT192 (AAC67784)	hypoth, 257aa	1e-120	2e-33, 231aa	*Caenorhabditis briggsae *(1.9, 1572aa)
CT226 (AAC67818)	hypoth, 176aa	4e-72	2e-12, 166aa	*Cryptosporidium parvum *(0.50, 1366aa)
CT227 (AAC67819)	hypoth, 133aa	4e-46	2e-04, 138aa	*Symbiobacterium thermophilum *(8.2, 143aa)
CT228 (AAC67820)	hypoth, 196aa	5e-76	4e-28, 210aa	*Campylobacter jejuni *(2.5, 176aa)
CT229 (AAC67821)	hypoth, 215aa	2e-86	4e-28, 214aa	*Bos taurus *(0.006, 1976aa)
CT249 (AAC67842)	hypoth, 116aa	6e-41	3e-20, 115aa	No other BLAST hits
CT300 (AAC67893)	hypoth, 115aa	2e-44	2e-05, 127aa	*Pan troglodytes *(6.3, 294aa)
CT326.2 (AAC67924)	hypoth, 60aa	2e-28	3e-04	No other BLAST hits
CT345 (AAC67940)	hypoth, 121aa	2e-52	5e-24, 121aa	*Haemophilus ducreyi *(4.7, 221aa)
CT357 (AAC67953)	hypoth, 110aa	8e-30	9e-07, 106aa	No other BLAST hits
CT358 (AAC67954)	hypoth, 178aa	2e-79	8e-31, 170aa	*Anabaena variabilis *(0.079, 185aa)
CT360 (AAC67956)	hypoth, 208aa	2e-94	2e-46, 212aa	*Caenorhabditis Elegans *(0.052, 728aa)
CT694 (AAC68289)	hypoth, 323aa	2e-177	8e-92, 334aa	*Psychrophila sp*. (0.56, 335aa)

These proteins are uniquely found in species belonging to the *Chlamydia *genus and are absent in the *Chlamydophila *and *Protochlamydia*.

**Table 4:** Listing and Characteristics of *Chlamydophila*-specific proteins

Protein Loci	Protein Name/Size	C. cav	C. abo	C. pne	Next Best Hit
CCA00062 (AAP04814)	YwbM, 463aa	0	6e-113, 501aa	1e-14, 382aa	*Aspergillus nidulans *(0.56, 182aa)
CCA00177 (AAP04928)	hypoth, 367aa	0	1e-146, 370aa	7e-35, 366aa	*Tetraodon nigroviridis *(2.7, 659aa)
CCA00189 (AAP04940)	hypoth, 316aa	0	2e-111, 316aa	3e-60, 312aa	*Novosphingobium aromaticivorans *(0.010, 331aa)
CCA00222 (AAP04973)	put. mb. protein, 123aa	1e-55	6e-22, 113aa	3e-05, 110aa	*Campellolebias dorsimaculatus*, (0.04, 348aa)
CCA00246 (AAP04997)	put. lipoprotein, 313aa	0	6e-158, 314aa	3e-96, 317aa	No other BLAST hits
CCA00261 (AAP05012)	put. mb. protein, 1044aa	0	0, 1105aa	3e-64, 1033aa	*Capsicum annuum *(0.18, 576aa)
CCA00296 (AAP05045)	hypoth, 448aa	0	4e-74, 442aa	2e-35, 445aa	*Ralstonia eutropha *(1.6, 556aa)
CCA00299 (AAP05048)	hypoth, 288aa	5e-146	4e-96, 288aa	3e-45, 289aa	*Neurospora crassa *(1.4, 541aa)
CCA00352 (AAP05100)	hypoth, 197aa	3e-95	2e-60, 204aa	5e-12, 212aa	*Cryptophlebia leucotreta *(0.016, 395aa)
CCA00353 (AAP05101)	hypoth, 186aa	3e-85	3e-60, 205aa	1e-11, 175aa	*Mus musculus *(0.12, 718aa)
CCA00360 (AAP05108)	put. inner mb. protein, 97aa	4e-47	4e-28, 104aa	1e-04, 101aa	*Arabidopsis thaliana *(0.58, 513aa)
CCA00361 (AAP05109)	put. inner mb. protein,, 110aa	4e-57	2e-42, 110aa	0.014, 111aa	*Bacillus licheniformis *(1.3, 416aa)
CCA00434 (AAP05180)	put. inner mb. protein, 451aa	0	4e-125, 435aa	6e-34, 424aa	other *Chlamydophila *hits ; *Apis mellifera *(0.93, 1500aa)
CCA00497 (AAP05241)	hypoth, 471aa	0	3e-173, 471aa	1e-55, 515aa	*Trophyrema whipplei *(2.9, 735aa)
CCA00530 (AAP05273)	hypoth, 424aa	0	1e-32, 435aa	6e-26, 435aa	*Fusobacterium nucleatum *(3.2, 448aa)
CCA00575 (AAP05317)	put. lipoprotein, 294aa	3e-165	1e-97, 277aa	3e-59, 284aa	*Wolinella. succinogenes *(2.5, 329aa)
CCA00576 (AAP05318)	hypoth 170aa	7e-88	1e-43, 155aa	2e-18, 164aa	*Melanoplus sanguinipes *(0.36, 254aa)
CCA00578 (AAP05320)	put. lipoprotein, 280aa	1e-160	7e-08, 277aa	2e-05, 284aa	*Plasmodium falciparum *(1.3, 294aa)
CCA00621 (AAP05363)	PTMP, 108aa	3e-54	2e-33, 108aa	4e-09, 161aa	*Agrobacterium tumefaciens *(0.34, 279aa)
CCA00738 (AAP05479)	lipoprotein, 95aa	5e-35	3e-15, 99aa	0.003, 99aa	*Mycobacterium leprae *(8.5, 574aa)

These proteins are uniquely found in species belonging to *Chlamydophila *genus but they are absent in various *Chlamydia *and *Protochlamydia *species as well as all other groups of bacteria. Abbreviations: put. inner mb., putative inner membrane protein, PTMP, putative transmembrane protein.

**Table 5:** Chlamydial Specific Proteins with Gene Losses in Some Species

Loci and Accession Numbers	Loci and Accession Numbers	Loci and Accession Numbers	Loci and Accession Numbers	Loci and Accession Numbers	Loci and Accession Numbers
*Chlamydophila *and *Protochlamydia *proteins (absent in *Chlamydia*)	*Chlamydiales *specific proteins missing *C. abortus*	*Chlamydiaceae *proteins, missing in *C. trachomatis*	*Chlamydiaceae *proteins, missing in *C. pneumoniae*	*Chlamydiaceae *proteins, missing in *C. caviae*	*Chlamydiaceae *proteins, missing in *C. muridarum*

CCA00154 (AAP04906)	CT550 (AAC68152)	CCA00231 (AAP04982)	CT049 (AAC67640)	CT244 (AAC67837)	CCA00470 (AAP05215)
CCA00168 (AAP04919)		CCA00396 (AAP05142)	CT050 (AAC67641)	CT277 (AAC67870)	CCA00259 (AAP05010)
CCA00180 (AAP04931)		CCA00425 (AAP05171)	CT051 (AAC67642)		
CCA00266 (AAP05017)		CCA00538 (AAP05281)	CT214 (AAC67806)		
CCA00333 (AAP05081)		CCA00827 (AAP05568)	CT867 (AAC68465)		
CCA00495 (AAP05239)		CCA00910 (AAP05649)	CT868 (AAC68466)		
CCA00619 (AAP05361)					
CCA00733 (AAP05474)					
CCA00743 (AAP05484)					
CCA00855 (AAP05596)					

Loci and Accession Numbers	Loci and Accession Numbers	Loci and Accession Numbers	Loci and Accession Number		

*Chlamydiaceae *proteins, missing *C. abortus*	*Chlamydophila *proteins, missing *C. pneumoniae*	*Chlamydia *proteins also in *C. pneumoniae*	*Chlamydiales, Rickettsiales *and plastids		

CT444.1 (AAC68048)	CCA00075 (AAP04827)	CT785 (AAC68380)	CT065 (O84068) CT495 (O84502)		
CT702 (AAC68297)			CT065 (O84068) CT495 (O84502)		

These chlamydiae-specific proteins are either missing in some species from particular groups or show unexpected presence in some other groups. The E values for these proteins are not listed.

**Table 6:** Stage-specific Expression of some of Chlamydiae-specific Proteins

Stage of Expression	*Chlamydiales*-specific Proteins	*Chlamydiaceae*-specific Proteins	*Chlamydia*-specific Proteins	Lost or Transferred Genes
I-Early (6–24 hpi)		CT233		
II-Midlate (12–24 hpi)	CT053 CT635	CT289 CT671	CT360	CT550
	CT066 CT666	CT372 CT870		
	CT273 CT667	CT377 CT871		
	CT504 CT670	CT413 CT872		
	CT590 CT734	CT618 CT874		
		CT668		
III-Midlate (18–24 hpi)		CT142		CT049
		CT143		CT051
		CT681		
		CT713		
IV-Late (24–36 hpi)	CT016	CT005 CT552	CT249	CT050
	CT017	CT046 CT565		CT214
	CT181	CT080 CT578	CT694	CT702
	CT443	CT082 CT579		
	CT546	CT083 CT620		CT868
		CT147 CT695		
		CT288 CT712		
		CT365 CT848		
		CT392 CT869		
		CT442		
		CT444		

The temporal expression patterns of these proteins is based upon the microarray analysis of Nicholson et al [63]. According to the microarray profiles, significant differences were seen in the expression pattern of genes for these proteins at 4 different time points (6–24 hours post infection, 12–24 h.p.i., 18–24 h.p.i., and 24–36 h.p.i.) which correspond to different phases of the chlamydial developmental cycle where key phenotypic changes take place. It is of interest that the majority of the temporally regulated *Chlamydiales *and *Chlamydiaceae*-specific genes are expressed between 12–24 h.p.i. and 24–36 h.p.i. Such coordinate regulation suggests they play essential roles during these phases of the chlamydial cell cycle. Other chlamydiae-specific proteins which are not listed in this Table were not found to show significant differences in their temporal expression in the study by Nicholson et al. [63]. Abbreviation; h.p.i., hours post infection.

**Table 7:** Sequence Divergence of Some (A) *Chlamydiales*-specific vs (B) Broadly-Distributed Proteins

**A**						
Protein Loci/Identity	C. tra	C. mur	C. pneu	C. cav	C. abo	Protochlam

CT007	316/316 (100%)	273/316 (86%)	184/316 (58%)	185/316 (58%)	183/316 (57%)	48/316 (23%)
CT016	242/242 (100%)	205/242 (84%)	146/246 (59%)	153/245 (62%)	148/245 (60%)	83/246 (33%)
CT017	433/433 (100%)	358/440 (81%)	224/401 (55%)	241/432 (55%)	248/432 (57%)	123/415 (29%)
CT021	247/247 (100%)	184/247 (74%)	102/244 (41%)	115/244 (47%)	108/245 (44%)	73/254 (28%)
CT031	100/100 (100%)	84/100 (84%)	65/100 (65%)	63/100 (63%)	63/100 (63%)	36/95 (37%)
CT038	116/116 (100%)	95/114 (83%)	55/112 (49%)	52/98 (53%)	49/98 (50%)	34/94 (36%)
Average	100%	81.5%	55%	56%	55%	31%

**B**						

Protein Loci/Identity	C. tra	C. mur	C. pneu	C. cav	C. abo	Protochlam

CT008 Ribo. HII	300/300 (100%)	257/301 (85%)	186/301 (61%)	185/300 (61%)	185/300 (61%)	139/301 (45%)
CT009 HthTR	152/152 (100%)	133/151 (87%)	105/143 (69%)	101/143 (66%)	105/143 (69%)	47/162 (28%)
CT013 Cyt. Ox. I	446/446 (100%)	406/445 (91%)	317/448 (70%)	325/446 (72%)	328/446 (73%)	195/465 (41%)
CT015 PhoH	434/434 (100%)	394/434 (90%)	314/424 (72%)	339/430 (78%)	338/430 (77%)	218/433 (49%)
CT019 IleRS	1036/1036 (100%)	923/1036 (89%)	715/1043 (68%)	729/1043 (69%)	729/1043 (69%)	575/1038 (55%)
CT022 Ribo. L31	108/108 (100%)	103/108 (95%)	90/109 (81%)	87/208 (80%)	86/108 (79%)	67/117 (57%)
CT034 YtfF	341/341 (100%)	279/341 (81%)	183/338 (53%)	203/338 (59%)	203/339 (59%)	94/281 (27%)
Average	100%	88%	67%	69%	69%	43%

The pair-wise amino acid sequence identity was determined for various chlamydial species for six *C. trachomatis *proteins that are specific for Chlamydiales (A) and seven other proteins that are broadly distributed in bacteria (B). The numbers in these Tables indicate the length over which the two sequences match each other as well as the % amino acid identity between the two sequences.

The abbreviations in protein names in part (B) are as follows: Ribo. HII, Ribonuclease HII; HthTR, Helix-turn-helix transcriptional regulator; Cyt. Ox. I, Cytochrome oxidase subunit I; PhoH, PhoH-ATPase; IleRS, Isoleucyl tRNA synthetase; Ribo. L31, Ribosomal L31 protein; YtfF, YytF-cationic amino acid transporter.

**Table 8:** Summary chart of the total unique proteins found for different chlamydiae lineage

Lineages	Number of unique ORFs (proteins)
***Chlamydiales ***(*C. trachomatis, C. pneumoniae, C. caviae, C. muridarum, C. abortus, P. amoebophila*)	**59**
***Chlamydiaceae ***(*C. trachomatis, C. pneumoniae, C. caviae, C. muridarum, C. abortus*)	**79**
***Chlamydophila ****(C. pneumoniae, C. caviae, C. abortus*)	**20**
***Chlamydia ***(*C. trachomatis, C. muridarum*)	**20**
***P. amoebophila***	**445**
